# Comparative ^1^H-NMR profiling of plasma and serum metabolites in giraffes and white rhinoceroses

**DOI:** 10.1007/s11306-026-02535-0

**Published:** 2026-09-23

**Authors:** G. Osthoff, S. Mason, L. Schmidt, A. Tordiffe, F. Deacon

**Affiliations:** 1 Department of Microbiology and Biochemistry, University of the Free State, Bloemfontein, South Africa; 2 Biomedical and Molecular Metabolism Research (BioMMet), Faculty of Natural and Agricultural Sciences, North-West University, Potchefstroom, South Africa; 3 Department of Paraclinical Sciences, Faculty of Veterinary Science, University of Pretoria, Pretoria, South Africa; 4 Department of Animal, Wildlife and Grassland Sciences, University of the Free State, Bloemfontein, 9300 South Africa

**Keywords:** Giraffe, White rhinoceros, EDTA plasma, Serum, Metabolomics, Nuclear magnetic resonance

## Abstract

**Background:**

Blood metabolite profiling can provide descriptive information on physiological variation in wildlife, but interpretation is sensitive to sampling design, specimen matrix and analytical batch.

**Objectives:**

To characterise quantified blood metabolites in apparently healthy giraffes and white rhinoceroses, examine sex-associated differences within each species, and conduct an exploratory matrix-matched interspecies comparison.

**Methods:**

Proton nuclear magnetic resonance spectra were available for 24 valid giraffe sampling occasions from 19 animals and for 45 white rhinoceroses. Metabolites were quantified jointly from the original spectra. Design-defined comparisons used log₂-transformed concentrations, Welch tests, Benjamini–Hochberg false-discovery correction, effect sizes and permutation, rank-based, leave-one-out and annotation-confidence sensitivity analyses.

**Results:**

No sex-associated metabolite differences were detected among the 2017 Rooipoort giraffe serum samples. Nine metabolites met the primary false-discovery criterion in the rhinoceros sex comparison, although only N-acetylglucosamine showed consistently strong evidence across all sensitivity analyses. In the comparison of six verified giraffe and 45 rhinoceros EDTA-plasma samples, 36 of 50 metabolites differed by the primary analysis; 28 formed a conservative core supported by all statistical tests and all leave-one-giraffe-out analyses. The core remained significant after adjustment for sex. Collection year was completely confounded with serum versus plasma in giraffes and was not tested inferentially.

**Conclusions:**

The two study groups had markedly different measured plasma metabolite profiles, with a coherent difference among branched-chain amino-acid-related metabolites. However, species was inseparable from NMR acquisition batch, and the small giraffe group limits biological and pathway-level inference. The concentrations provide exploratory baseline data rather than formal reference intervals.

**Supplementary Information:**

The online version contains supplementary material available at https://doi.org/10.1007/s11306-026-02535-0.

## Introduction

Circulating metabolites integrate contributions from multiple organs and can reflect genetic background, physiological state, nutrition, microorganisms, xenobiotic exposure and disease. Blood metabolomics has consequently been applied to spontaneous animal disease (Tran et al., 2020), nutrition (Tordiffe et al., 2016; Tordiffe & Mienie, 2019), and physiological variation in wildlife (Wood et al., 2022; Corder et al., 2023). The interpretation of such profiles nevertheless depends on the biological question, the sampled population, specimen handling and the analytical platform.

Metabolomic data remain comparatively scarce for wildlife. Studies have described serum amino acids and fatty acids in cheetahs (Tordiffe et al., 2016; Tordiffe & Mienie, 2019), blood and other biofluids in giant pandas and African savanna elephants (Zhu et al., 2020; Wood et al., 2022; van Zyl et al., 2023), milk in dromedary, giraffe and white rhinoceros (Osthoff et al., 2025) and metabolic differences associated with disease or taxon in black and Sumatran rhinoceroses (Watanabe et al., 2016; Corder et al., 2023). These investigations illustrate the potential value of species-specific baseline data, but also show that metabolite concentrations are affected by sex, health, diet, management and analytical method.

Giraffes (*Giraffa camelopardalis*) and southern white rhinoceroses (*Ceratotherium simum*) are evolutionarily distant large herbivores with different digestive strategies: giraffes are foregut-fermenting ruminant browsers, whereas white rhinoceroses are hindgut-fermenting grazers (Mitchell, 2021; Endo et al., 1999). Their measured blood profiles may therefore differ because of species physiology, diet and microbial fermentation. An interspecies comparison can identify candidate biochemical patterns for future targeted investigation, but cannot by itself establish causal mechanisms or species reference intervals.

Apparently healthy animals managed under free-ranging or semi-extensive conditions are valuable sources of otherwise scarce descriptive data. Such datasets may guide future study design and analytical verification in conservation medicine and environmental metabolomics (Bundy et al., 2009; Cooke et al., 2013; Lankadurai et al., 2013). They should not be described as clinical reference intervals unless the sampling design and analysis meet dedicated veterinary reference-interval standards (Friedrichs et al., 2012). The limited sample sizes available for many wildlife species make effect estimation, multiplicity control and transparent sensitivity analyses particularly important.

The objectives of this study were therefore to describe quantified blood metabolites in apparently healthy giraffes and white rhinoceroses, test for sex-associated differences within design-compatible sample groups, and compare the species using EDTA plasma only. A collection-year comparison in giraffes was considered during data audit but was restricted to descriptive reporting because year was completely confounded with serum versus EDTA plasma.

## Materials and methods

### Animal handling and blood collection

Blood was collected from 45 apparently healthy, free-ranging white rhinoceroses (31 females and 14 males) during routine management procedures on a single semi-extensive wildlife property in the Northern Cape Province of South Africa from 20 to 24 May 2019. The rhinoceroses ranged on natural vegetation supplemented with lucerne during dry periods. Animals were immobilised for veterinary examination and pregnancy assessment using etorphine (6 mg), hyaluronidase (2500 IU) and azaperone (40 mg) administered by remote injection. Anaesthesia was antagonised with diprenorphine. Blood was collected from a posterior auricular vein through a 12-gauge needle into 8.5 mL K₃-EDTA tubes. Samples were centrifuged within 2 h of collection at 1200 rpm for 10 min, and plasma aliquots were stored at − 80 °C until analysis.

Giraffe concentration data were available for 24 valid sampling occasions from 19 apparently healthy, free-ranging giraffes. In 2017, serum was available from 18 sampling occasions: 16 giraffes at Rooipoort Nature Reserve, Kimberley (10 females and six males), and two females at Sandveld Nature Reserve, Hoopstad. In 2018, EDTA plasma was obtained from six giraffes at Rooipoort (two females and four males); five of these animals also had usable 2017 serum data. Giraffes were immobilised as described by Deacon et al. (2022). Briefly, thiafentanil (8–10 mg) was administered by remote injection, followed by butorphanol (25 mg) after recumbency. Blood was collected from a jugular vein through a 12-gauge needle. Plain red-top tubes were used in 2017 and purple-top K₃-EDTA tubes in 2018. Serum or plasma was separated within 2 h by centrifugation at 1,200 rpm for 10 min and stored at − 80 °C.

### Preparation of blood samples for ^1^H-NMR analysis

Samples were thawed at room temperature. Amicon Ultra 2 mL centrifugal units with a 10 kDa membrane filter (Merck, Darmstadt, Germany) were rinsed five times with distilled water by centrifugation at 6,000 × g for 5 min in a swing-bucket centrifuge to remove residual glycerol. One millilitre of serum or plasma was then filtered at 4,500 × g for 30 min to remove proteins and other macromolecules. A 300 µL aliquot of filtrate was mixed with 300 µL NMR buffer comprising 0.075 M sodium phosphate in deuterium oxide, adjusted to pH 7.40 and containing trimethylsilyl-2,2,3,3-tetradeuteropropionic acid (TSP) as the internal standard. After vortex mixing for 5 s and centrifugation at 4,500 × g for 30 min, 540 µL of supernatant was transferred to a capped 5 mm glass NMR tube.

### ^1^H-NMR acquisition

Spectra were acquired using a Bruker Avance III HD 500 MHz NMR spectrometer fitted with a triple-resonance inverse TXI ¹H{¹⁵N, ¹³C} probe and x, y and z gradient coils. One-dimensional ^1^H spectra were acquired as 128 transients into 32 K data points using a 12,000 Hz spectral width, 2.72 s acquisition time, receiver gain of 64 and sample temperature of 300 K. Water resonance was presaturated using a one-dimensional NOESY sequence during a 4 s relaxation delay, with a 90° excitation pulse of 8 µs. Shimming was performed automatically on the deuterium signal. Fourier transformation and initial phase and baseline correction were performed in Bruker TopSpin version 3.7. Spectral resolution was checked manually by confirming that the full width at half maximum of the TSP peak was < 1 Hz.

The giraffe spectra were acquired on 19 November 2018 (SM-20181119 series) and the rhinoceros spectra on 6 October 2021 (CRS-20211006 series). All biological samples were analysed in one dimension. A species-specific pooled representative sample was also analysed by J-resolved (JRES) and correlation spectroscopy (COSY) to support metabolite annotation. The giraffe and rhinoceros pools were separate and were used for annotation, not as common cross-batch quality-control material.

### Joint spectral processing, metabolite annotation and quantification

In November 2024, the original giraffe and rhinoceros one-dimensional spectra were reprocessed and quantified together using Bruker AMIX version 3.9.14. Metabolites were annotated against a pure-compound one- and two-dimensional ^1^H-NMR spectral library and the species-specific JRES and COSY spectra. Isolated, non-overlapping resonances were selected for quantification relative to the TSP internal standard, and concentrations were reported in micromoles per litre (µmol/L). For rhinoceros W46, the repeat acquisition CRS-20211006-50 was used for quantification.

Metabolite identity was classified according to the Metabolomics Standards Initiative (Sumner et al., 2007). Level 1 assignments were confirmed against reference standards and supported by two-dimensional COSY spectra; level 2 assignments were putatively annotated from one-dimensional resonances when two-dimensional confirmation was not possible. Resonances, chemical shifts, Human Metabolome Database identifiers and identity levels are provided in Supplementary Table S1. When no discernible resonance was present, the result was left blank rather than assigned a concentration of zero.

### Data curation and analysis populations

**Fig. 1:**
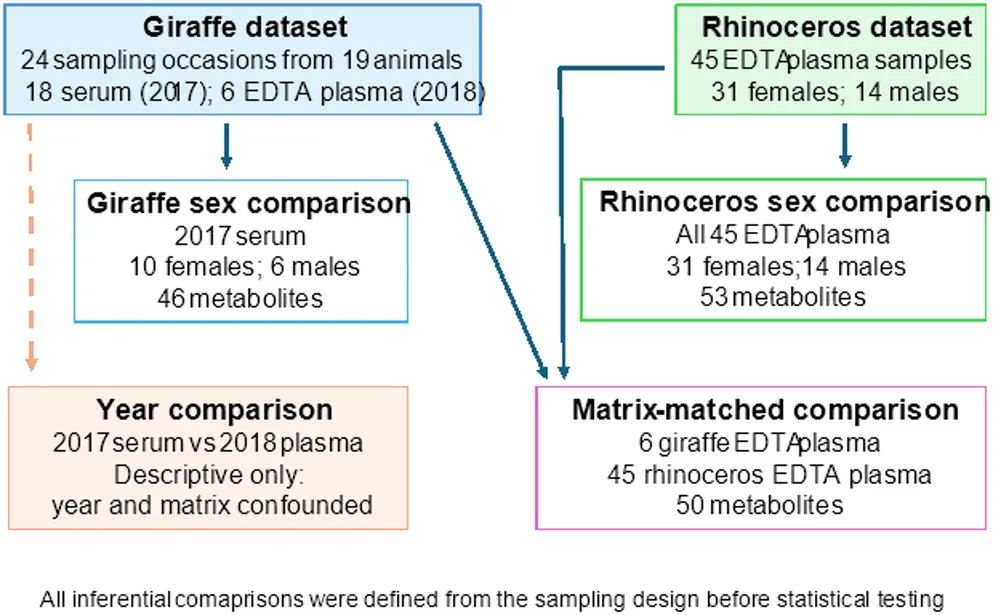
Sample curation and design-defined analysis populations. F7B was excluded as a duplicate analytical record of F7A, and BM4 was excluded because its biological identity could not be verified. The 2017–2018 giraffe comparison was not tested inferentially because year and specimen matrix were completely confounded

Biological sample identifiers were reconciled with the original collection records and NMR acquisition folders before analysis (Fig. 1). Two samples that had been flagged during earlier work, were retained in the primary dataset and excluded together in a sensitivity analysis.

Three primary contrasts were defined from the sampling design before statistical testing. The giraffe sex comparison used only 2017 Rooipoort serum samples (10 females and six males), thereby holding collection year, reserve and specimen matrix constant. The white-rhinoceros sex comparison included all 45 EDTA-plasma samples. The interspecies comparison used only matrix-matched EDTA plasma from the six verified 2018 giraffes and the 45 rhinoceroses. The 2017–2018 giraffe comparison was restricted to descriptive reporting because collection year was completely confounded with serum versus EDTA plasma. No statistical batch correction was attempted for the interspecies contrast because species and NMR acquisition batch were also perfectly confounded.

Ca-EDTA and Mg-EDTA were treated as technical signals and excluded from biological hypothesis testing. For the interspecies analysis, cholic acid, glycocholic acid and N, N-dimethylformamide were excluded because they were not detected in any giraffe sample, and N, N-dimethylglycine was excluded because it was not quantified in the rhinoceros dataset. Fifty metabolites were therefore directly comparable between the matrix-matched species groups.

### Statistical analysis

Concentration values of zero and blank cells were treated as missing; no missing value was set to zero or imputed. A metabolite was eligible for a contrast when positive concentrations were available for at least four samples in each group. Concentrations were transformed to log₂ before parametric testing. Pareto scaling was not applied because the primary analyses were conducted separately for each metabolite and effect estimates were intended to remain interpretable on the concentration scale.

The primary test for each metabolite was a two-sided Welch independent-samples t-test, which does not assume equal group variances (Welch, 1947). The Benjamini–Hochberg procedure was applied separately to the family of metabolites within each predefined contrast, and a false-discovery-rate-adjusted p value (q value) < 0.05 was considered statistically significant (Benjamini & Hochberg, 1995). No minimum fold-change threshold was imposed. Effects were expressed as ratios of group geometric means, calculated by exponentiating the mean difference on the log_2_ scale. Nominal 95% confidence intervals for the log₂ difference and Hedges’ g (Hedges, 1981) were also calculated. Arithmetic means on the concentration scale were retained for descriptive tables.

Robustness was evaluated using an equal-variance Student t-test, a two-sided Mann–Whitney U test and a Monte Carlo studentized permutation test based on the absolute Welch t statistic (50,000 label permutations). Benjamini–Hochberg correction was applied separately to each sensitivity test. Stability was assessed by repeating the complete Welch analysis and FDR correction after omitting each rhinoceros in turn and, for the interspecies analysis, after omitting each of the six giraffes in turn. Analyses restricted to MSI level 1 metabolites assessed sensitivity to annotation confidence. The giraffe sex analysis was additionally repeated after two female giraffe samples were excluded together in a sensitivity analysis and adding the two Sandveld females.

Because the species groups had different sex ratios, the interspecies comparison was also repeated using a linear model of log_2_ concentration with species and sex as predictors. Heteroscedasticity-consistent HC3 standard errors were used (MacKinnon & White, 1985), followed by Benjamini–Hochberg correction across the 50 species coefficients. This model was treated as a sensitivity analysis because only six giraffe plasma samples were available.

For the rhinoceros sex contrast, ‘strongest evidence’ required q < 0.05 with Welch, permutation and Mann–Whitney tests and significance in all 45 leave-one-out runs; ‘moderate evidence’ required Welch and permutation q < 0.05 and significance in at least 30 of 45 leave-one-out runs; other Welch-significant findings were labelled ‘threshold-sensitive’. For the interspecies contrast, the conservative core required q < 0.05 with all three tests and significance in all six leave-one-giraffe-out runs. Other Welch-significant findings were labelled sensitivity-dependent. These categories were descriptive summaries of robustness rather than additional hypothesis tests.

The definitive analysis did not use PLS-DA, OPLS-DA, VIP-based feature selection, random-forest classification or pathway enrichment as inferential evidence. Analyses were performed in Python 3.12.13 using NumPy 2.3.5, pandas 2.2.3 and SciPy 1.17.0. Fixed random seeds were used for permutation procedures, and the analysis script and complete metabolite-level outputs were retained for reproducibility.

##  Results

### Data curation and metabolite coverage

After exclusion of two female giraffe samples the definitive giraffe dataset comprised 24 sampling occasions from 19 animals: 18 samples collected in 2017 (16 at Rooipoort and two at Sandveld) and six collected at Rooipoort in 2018. Five giraffes were sampled in both years. The rhinoceros dataset comprised 45 EDTA-plasma samples collected on a single property over five days in May 2019 (Fig. 1; Table 1).

**Table 1:** Design-defined analysis populations and primary outcomes

Analysis	Samples	Metabolites tested	Welch FDR q < 0.05	Interpretation
Giraffe sex	2017 Rooipoort serum: 10 F, 6 M	46	0	No sex-associated differences detected
Giraffe year	2017 serum vs. 2018 EDTA plasma	Not tested	Not tested	Year and specimen matrix completely confounded
Rhinoceros sex	EDTA plasma: 31 F, 14 M	53	9	1 strongest, 2 moderate and 6 threshold-sensitive findings
Interspecies	EDTA plasma: 6 giraffe, 45 rhinoceros	50	36	28-metabolite conservative core

*F* female, *M* male, *FDR* Benjamini–Hochberg false-discovery rate. The giraffe year comparison was retained for descriptive reporting only

After exclusion of the Ca-EDTA and Mg-EDTA signals, 54 biological metabolites were represented across the two concentration tables. Fifty-one were detected in at least one giraffe sample and 53 in rhinoceros samples. Cholic acid, glycocholic acid and N, N-dimethylformamide were not detected in any giraffe sample, whereas N, N-dimethylglycine was not included in the rhinoceros concentration table. Blank cells were treated as non-detections and were not converted to zero or imputed.

### Sex comparison in giraffes

The primary giraffe sex comparison included the 2017 Rooipoort serum samples from 10 females and six males. Forty-six metabolites were measurable in both groups. No metabolite differed at the unadjusted 0.05 level by Welch testing; the smallest p value was for 3-hydroxyisobutyric acid (female-to-male fold change 1.26, *p* = 0.070, q = 0.895). Studentized permutation testing likewise identified no FDR-significant metabolites.

The conclusion was unchanged when F6 and F8 were excluded, when F7B was used in place of F7A, or when the two Sandveld females were added. The latter analysis produced five nominal p values below 0.05, but none remained significant after FDR correction. Thus, no sex-associated differences were detected in the 2017 giraffe serum dataset; this should not be interpreted as evidence that the sexes were metabolically equivalent.

### Giraffe collection year and specimen matrix

The 18 giraffe samples collected in 2017 were serum, whereas the six collected in 2018 were EDTA plasma. Collection year and specimen matrix were therefore completely confounded, and no inferential 2017–2018 comparison or pathway-enrichment analysis was performed. Five animals had samples in both years, but their paired changes could not separate a year effect from a matrix effect.

The non-detection pattern reinforced this limitation. Ca-EDTA, Mg-EDTA, methylamine, dimethylamine, sarcosine and trimethylamine were blank in all 18 serum samples but detected in all six EDTA-plasma samples. Aspartic acid was detected in three of 18 serum samples and all six plasma samples. These observations were treated descriptively and not interpreted as evidence of drought, diet or a temporal effect.

### Sex comparison in white rhinoceroses

**Fig. 2:**
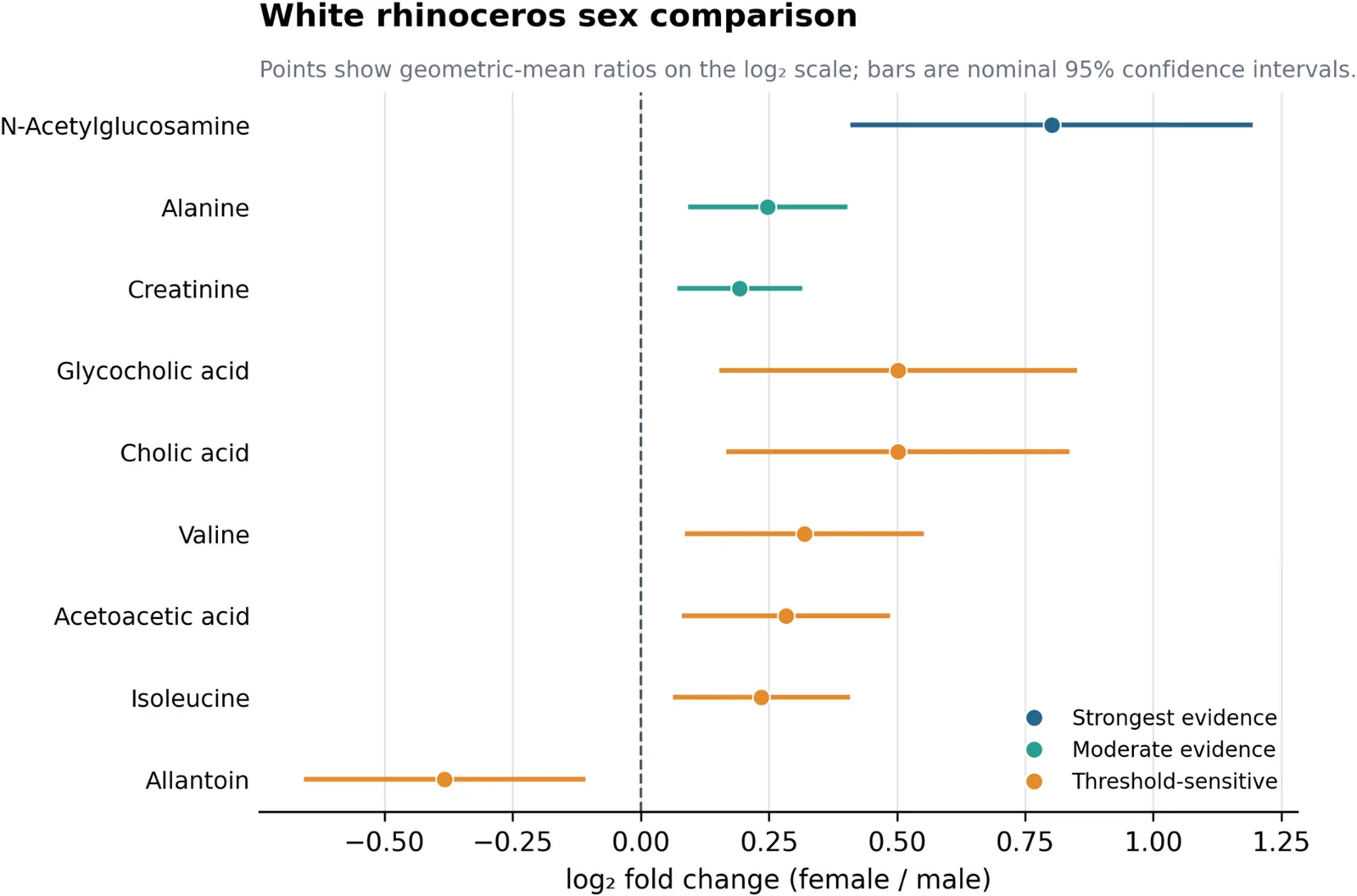
Effect estimates for the nine metabolites meeting the primary Welch-FDR criterion in the white-rhinoceros sex comparison. Points are female-to-male log₂ fold changes and horizontal lines are nominal 95% confidence intervals. Colours indicate the prespecified descriptive robustness categories

Among 53 biological metabolites, nine met the primary Welch-test criterion after Benjamini–Hochberg correction (Table 2; Fig. 2). Female rhinoceroses had higher N-acetylglucosamine, creatinine, alanine, cholic acid, glycocholic acid, acetoacetic acid, isoleucine and valine, and lower allantoin than males.

**Table 2:** Metabolites meeting the primary Welch-FDR criterion in the white-rhinoceros sex comparison

Metabolite	MSI	Female mean	Male mean	FC F/M	Welch q	Evidence
N-Acetylglucosamine	1	13.01	7.48	1.74	0.014	Strongest
Creatinine	2	17.19	14.90	1.14	0.040	Moderate
Alanine	1	63.90	53.65	1.19	0.040	Moderate
Cholic acid	2	1.61	1.14	1.42	0.048	Threshold-sensitive
Glycocholic acid	2	1.32	0.94	1.42	0.048	Threshold-sensitive
Allantoin	2	11.19	14.27	0.77	0.048	Threshold-sensitive
Acetoacetic acid	2	1.69	1.40	1.22	0.048	Threshold-sensitive
Isoleucine	1	14.14	11.97	1.18	0.048	Threshold-sensitive
Valine	1	43.03	34.73	1.25	0.048	Threshold-sensitive

Means are arithmetic concentrations in µmol/L. FC is the female-to-male ratio of geometric means. MSI, Metabolomics Standards Initiative identity level. Robustness categories incorporate permutation, Mann–Whitney and leave-one-out sensitivity analyses; complete results are provided in the Supplementary Information

The strength of evidence was not uniform. N-acetylglucosamine remained significant with Welch, Mann–Whitney and studentized permutation tests and in all 45 leave-one-out analyses. Alanine and creatinine remained significant in the permutation analysis and in 32 and 33 leave-one-out runs, respectively, although their Mann–Whitney q values were 0.055. The remaining six metabolites were close to the FDR threshold and did not remain significant after permutation or Mann–Whitney correction. The equal-variance t-test identified 11 FDR-significant metabolites, whereas restriction to 28 MSI level 1 assignments retained N-acetylglucosamine and alanine.

### Matrix-matched interspecies comparison

**Fig. 3:**
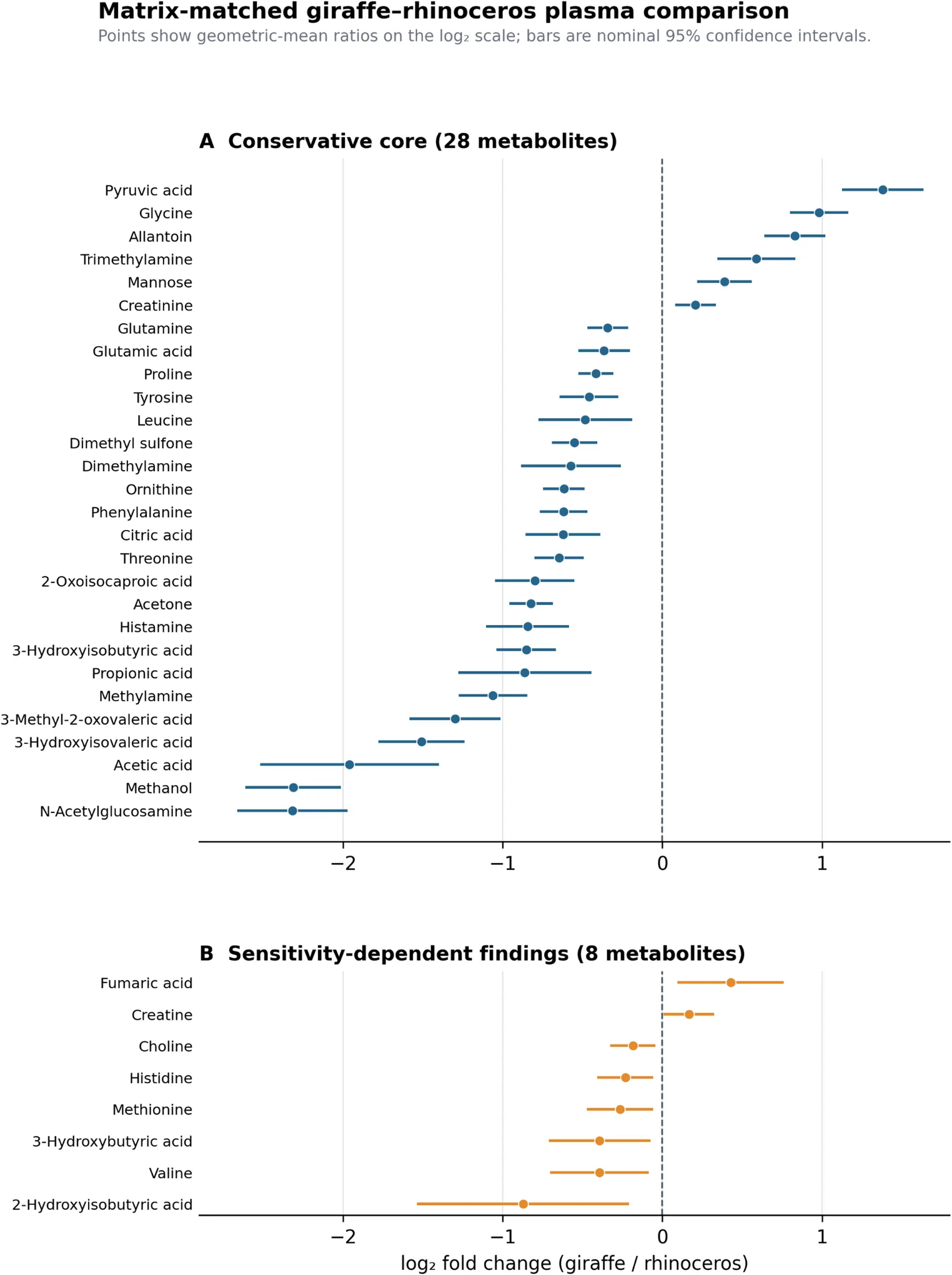
Effect estimates for the 36 metabolites meeting the primary Welch-FDR criterion in the matrix-matched interspecies comparison. Points are giraffe-to-rhinoceros log₂ fold changes and horizontal lines are nominal 95% confidence intervals. Panel A shows the 28-metabolite conservative core; panel B shows the eight sensitivity-dependent findings

The interspecies analysis compared EDTA plasma from six verified 2018 giraffes with all 45 rhinoceroses. Fifty metabolites were directly comparable. Thirty-six were significant after Welch testing and FDR correction. Thirty-six also passed the studentized permutation analysis, 34 passed the Mann–Whitney analysis, and 29 remained FDR significant in all six leave-one-giraffe-out analyses. The intersection of these criteria defined a conservative core of 28 metabolites, including 15 MSI level 1 assignments (Table 3; Fig. 3A). Eight additional metabolites met the primary Welch-FDR criterion but were sensitivity-dependent (Table 4; Fig. 3B).

**Table 3:** Conservative core of the matrix-matched giraffe–rhinoceros EDTA-plasma comparison

Metabolite	MSI	Giraffe mean	Rhino mean	FC G/*R*	Welch q	Sex-adjusted q
Methanol	2	11.64	68.06	0.20	2.79E-19	1.93E-19
Acetone	2	0.94	1.72	0.57	1.12E-14	4.63E-14
Pyruvic acid	2	24.33	10.18	2.60	1.78E-10	3.72E-11
Allantoin	2	20.59	12.15	1.78	5.27E-09	1.43E-05
N-Acetylglucosamine	1	2.07	11.29	0.20	5.62E-09	5.45E-12
Threonine	1	23.92	38.09	0.64	1.34E-06	3.45E-09
Glycine	2	44.77	22.98	1.98	1.34E-06	5.45E-12
Proline	1	26.85	36.11	0.75	6.23E-06	4.15E-09
Ornithine	1	8.69	13.37	0.65	1.66E-05	1.48E-13
3-Hydroxyisovaleric acid	2	1.26	3.58	0.35	2.31E-05	3.66E-16
3-Hydroxyisobutyric acid	1	2.79	5.07	0.55	2.70E-05	3.35E-12
Phenylalanine	1	9.74	15.04	0.65	3.08E-05	9.70E-11
3-Methyl-2-oxovaleric acid	1	1.04	2.58	0.41	4.91E-05	1.25E-13
Dimethyl sulfone	2	11.71	17.19	0.68	9.37E-05	6.06E-11
Methylamine	2	0.67	1.40	0.48	9.37E-05	1.22E-14
Glutamine	1	23.11	29.64	0.79	9.37E-05	2.70E-05
Trimethylamine	2	1.40	0.99	1.50	1.65E-04	0.038
Acetic acid	2	29.73	117.0	0.26	2.60E-04	4.33E-09
Histamine	2	6.04	10.86	0.56	3.75E-04	7.11E-08
Mannose	1	7.94	6.18	1.31	4.58E-04	5.76E-05
2-Oxoisocaproic acid	1	2.13	3.70	0.57	4.93E-04	7.44E-11
Tyrosine	1	7.70	10.60	0.73	0.001	3.72E-06
Citric acid	1	41.36	63.38	0.65	0.001	6.72E-07
Glutamic acid	1	50.43	65.17	0.78	0.002	1.39E-06
Propionic acid	1	1.73	3.29	0.55	0.003	1.16E-04
Creatinine	2	18.83	16.47	1.16	0.005	0.008
Dimethylamine	2	1.26	1.86	0.67	0.008	4.28E-05
Leucine	1	16.33	22.72	0.72	0.011	2.85E-04

Means are arithmetic concentrations in µmol/L. FC is the giraffe-to-rhinoceros ratio of geometric means. Core metabolites had q < 0.05 with Welch, studentized permutation and Mann–Whitney tests and in all six leave-one-giraffe-out analyses. Sex-adjusted q values are from linear models with HC3 standard errors

**Table 4:** Sensitivity-dependent findings in the matrix-matched giraffe–rhinoceros EDTA-plasma comparison

Metabolite	MSI	Giraffe mean	Rhino mean	FC G/*R*	Welch q	Sex-adjusted q
Fumaric acid	2	1.00	0.89	1.34	0.020	0.054
Choline	1	9.32	10.64	0.88	0.021	0.013
Histidine	2	9.80	11.55	0.85	0.021	0.047
2-Hydroxyisobutyric acid	2	0.79	1.62	0.55	0.024	0.013
Methionine	1	12.14	14.64	0.83	0.025	0.004
Valine	1	30.40	40.45	0.76	0.025	0.010
3-Hydroxybutyric acid	1	29.97	41.29	0.76	0.026	0.020
Creatine	2	22.74	20.99	1.12	0.042	0.634

Means are arithmetic concentrations in µmol/L. FC is the giraffe-to-rhinoceros ratio of geometric means. These metabolites met the primary Welch-FDR criterion but did not satisfy every permutation, rank-based or leave-one-giraffe-out criterion. Fumaric acid and creatine did not remain significant after sex adjustment

Among the conservative-core metabolites, giraffe plasma had higher pyruvic acid (giraffe-to-rhinoceros fold change 2.60), glycine (1.98), allantoin (1.78) and trimethylamine (1.50). It had lower methanol (0.20), N-acetylglucosamine (0.20), acetic acid (0.26), 3-hydroxyisovaleric acid (0.35), 3-methyl-2-oxovaleric acid (0.41) and methylamine (0.48), among other differences.

Adjustment for sex retained 34 of the 36 primary findings; all 28 conservative-core metabolites remained FDR significant. Fumaric acid (sex-adjusted q = 0.054) and creatine (q = 0.634), both sensitivity-dependent in the primary analysis, did not remain significant after adjustment. The broad interspecies pattern was therefore not explained by the different sex ratios.

The giraffe and rhinoceros spectra were nevertheless acquired in separate analytical batches in 2018 and 2021, respectively, and no common pooled quality-control material was run across the batches. Although the spectra were reprocessed and quantified together in November 2024, species and acquisition batch remain inseparable. The results therefore describe differences between the two study groups and do not establish that every difference was biological.

## Discussion

Three principal findings emerged. First, no sex-associated metabolite difference was detected in the design-compatible giraffe serum comparison. Second, the rhinoceros sex analysis produced one particularly robust finding, two findings with moderate support and six threshold-sensitive findings. Third, the two matrix-matched species groups differed across a substantial proportion of the quantified metabolites. Interpretation is restricted to measured concentration patterns because the small giraffe group and complete confounding of species with acquisition batch preclude firm mechanistic pathway inference.

The absence of FDR-significant sex differences among giraffes is consistent with reports that sex effects are not universal across wildlife metabolomes (Wood et al., 2022). It should not, however, be interpreted as proof of equivalence. With only 10 females and six males, the study had limited ability to detect modest effects, and no biologically justified equivalence margin was specified. Reporting the null result remains important because the comparison addressed a predefined objective and held year, reserve and specimen matrix constant.

The white-rhinoceros sex comparison illustrates why multiplicity correction and sensitivity analysis are needed. Nine metabolites passed the primary Welch-FDR criterion, but only N-acetylglucosamine satisfied every robustness criterion. Alanine and creatinine had moderate support, while six results lay close to the FDR boundary. These observations justify cautious reporting of sex-associated concentration differences but do not provide robust evidence of altered bile-acid, androstenedione, pterin or folate pathways. The data represent circulating concentrations rather than pathway flux, and pathway-level inference based on a small number of overlapping metabolites would be unreliable.

The most coherent biochemical pattern in the interspecies comparison involved branched-chain amino-acid-related metabolites. Giraffe plasma had lower leucine, 2-oxoisocaproic acid, 3-methyl-2-oxovaleric acid, 3-hydroxyisobutyric acid and 3-hydroxyisovaleric acid; valine was also lower but was sensitivity-dependent. The concordant direction among these related compounds is consistent with a difference in the circulating branched-chain amino-acid profile or its catabolic products. It does not identify whether the difference arose from intake, ruminal or hindgut microbial metabolism, tissue use, clearance, fasting state or analytical batch.

Other differences were less readily reduced to single pathway explanations. Giraffe plasma contained more pyruvate but less citrate, while lactate and succinate did not differ significantly. Acetate and propionate were lower in giraffes, which is biologically compatible with differences between foregut and hindgut fermentation, diet and hepatic use of absorbed organic acids (Mitchell, 2021; Endo et al., 1999), but the study did not measure feed intake, fermentation products or sampling relative to feeding. Similarly, higher trimethylamine occurred alongside lower methylamine and dimethylamine, a weaker decrease in choline, and no difference in sarcosine. This mixed pattern does not support a simple increase or decrease in choline catabolism or one-carbon metabolism.

The non-detection of cholic acid, glycocholic acid and N, N-dimethylformamide in giraffe samples, and the absence of N, N-dimethylglycine from the rhinoceros concentration table, cannot be treated as quantitative interspecies differences. A blank indicates that no discernible resonance was quantified; it does not demonstrate biological absence. These analytes were therefore excluded from the matrix-matched comparison, and the available data cannot support conclusions about limited circulating bile acids in giraffes or altered one-carbon metabolism in white rhinoceroses.

Several study limitations remain. Only six verified giraffe plasma samples were available, although leave-one-giraffe-out analyses showed that the conservative core was not driven by a single animal. Giraffe and rhinoceros samples came from different populations, years and ecological settings, and the species groups had different sex ratios. Adjustment for sex did not materially change the conservative core, but unmeasured diet, fasting duration, handling and environmental factors remain potential explanations. Most importantly, all giraffe spectra were acquired in one analytical series and all rhinoceros spectra in another, without a shared cross-batch quality-control pool. The consistency of acquisition parameters and joint reprocessing support comparability, but they do not permit batch effects to be separated from species effects.

The quantified concentrations are useful as exploratory baseline data from apparently healthy animals and can inform future targeted studies. They are not formal clinical reference intervals, which would require a larger and more representative reference population, controlled pre-analytical procedures and dedicated reference-interval methods (Friedrichs et al., 2012). Future work should acquire both species prospectively using the same specimen matrix, balanced sex groups, contemporaneous acquisition, shared quality-control material and documented diet and sampling relative to feeding.

## Conclusions

No sex-associated differences were detected in the design-compatible giraffe serum comparison. Nine metabolites met the primary criterion in the white-rhinoceros sex comparison, but only N-acetylglucosamine was consistently supported by all sensitivity analyses. The matrix-matched giraffe and rhinoceros study groups differed across 36 of 50 quantified metabolites, with a conservative core of 28 and a coherent pattern among branched-chain amino-acid-related compounds. These findings support further targeted comparative work but do not establish pathway activation or species-specific mechanisms because species remained confounded with NMR acquisition batch.

## Supplementary Information

Below is the link to the electronic supplementary material.

Supplementary Material 1

Supplementary Material 2 Supplementary Table S1 provides the metabolite chemical shifts, structures, Human Metabolome Database identifiers and MSI identity levels. Additional supplementary tables provide the sample and NMR-folder audit, all giraffe sex results, the giraffe detection-by-year/matrix audit, all rhinoceros sex results, and all matrix-matched interspecies results, including robustness and sex-adjusted sensitivity analyses

## Data Availability

No datasets were generated or analysed during the current study.
