# Supplementary material for: Comparative 1H-NMR profiling of plasma and serum metabolites in giraffes and white rhinoceroses
Source: Metabolomics. 2026 Sep 23;22(5):159. doi: 10.1007/s11306-026-02535-0 (PMC13601079; doi:10.1007/s11306-026-02535-0)
Supplement: Supplementary file 1 [file 11306_2026_2535_MOESM1_ESM.pdf]

Table S1: Chemical information and shifts for representative ^{1}H-NMR spectra of pooled blood samples from giraffes (black) and rhinos (blue). Multiplicity: s − singlet; d − doublet; t – triplet; dd − double doublet; dt – double triplet; tt = triple triplet; q – quartet; p – pentet; h – hextet; m – multiplet; AB – AB system; BS – broad singlet. ND = not determined.

Peak assignment	Metabolite name (*MSI metabolite identity)	Chemical structure	HMDB ID	Chemical formula	δ (ppm), multiplicity (J coupling)	Chemical group
1	Cholic acid (level 2)	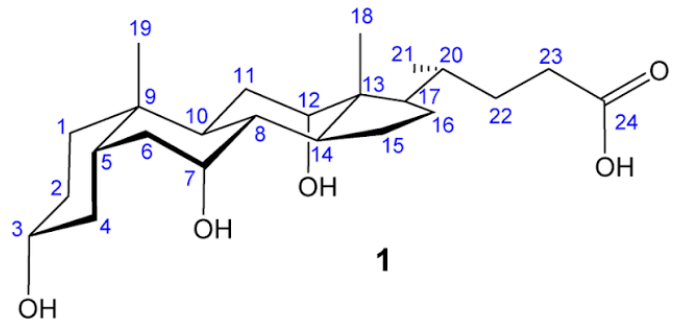 1	HMDB0000619	$C_{24}H_{40}O_5$	0.83 s	CH_3
2	Glycocholic acid (level 2)	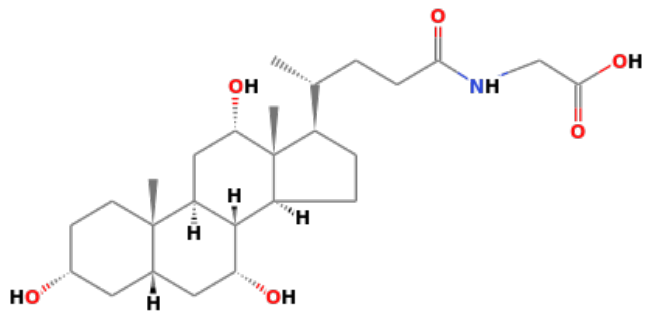	HMDB0000138	$C_{26}H_{43}NO_6$	0.84 s	CH_3
3	Isoleucine (level 1)	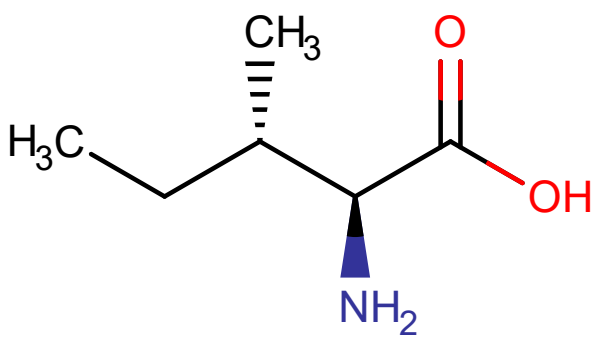	HMDB0000172	$C_6H_{13}NO_2$	0.94 t (J = 7.3 Hz)	CH_3
					1.01 d (J = 7.1 Hz)	CH_3
4	Leucine (level 1)	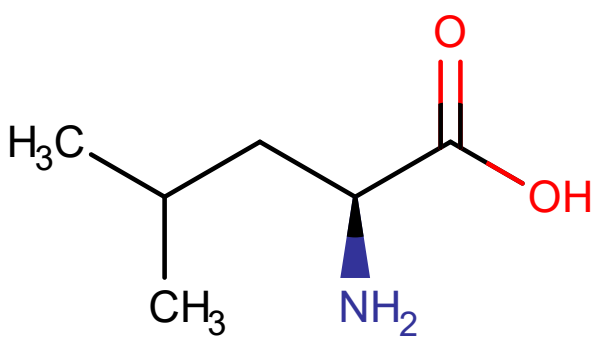	HMDB0000687	$C_6H_{13}NO_2$	0.96 dd (J = 6.2 Hz)	$(CH_3)_2$

Peak assignment	Metabolite name (*MSI metabolite identity)	Chemical structure	HMDB ID	Chemical formula	δ (ppm), multiplicity (J coupling)	Chemical group
5	Valine (level 1)	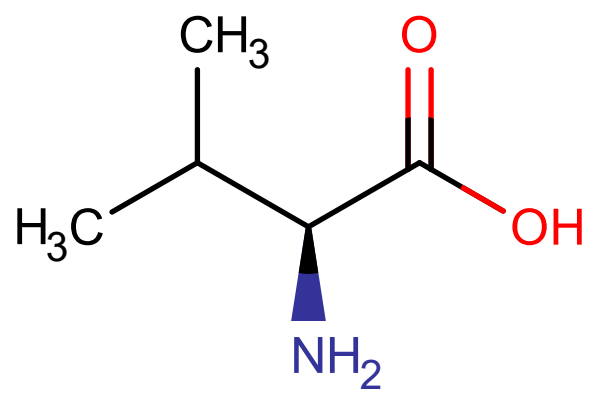	HMDB0000883	$C_5H_{11}NO_2$	0.99 d (J = 7.0 Hz)	CH_3
					1.04 d (J = 7.0 Hz)	CH_3
					2.28 m	CH_2
6	2-Hydroxybutyric acid (level 1)	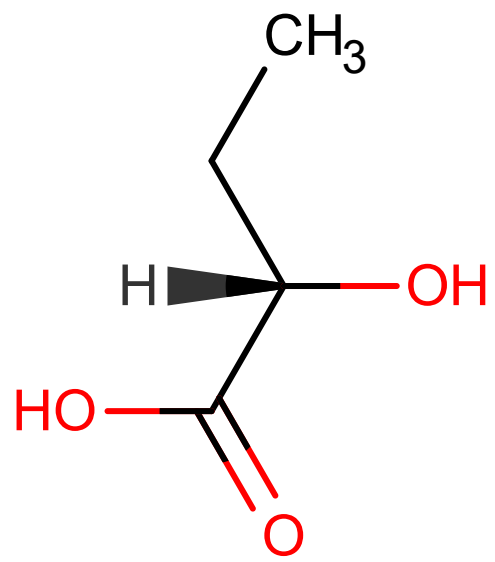	HMDB0000008	$C_4H_8O_3$	0.89 t (J = 7.3 Hz)	CH_3
7	3-Methyl-2-oxovaleric acid (level 1)	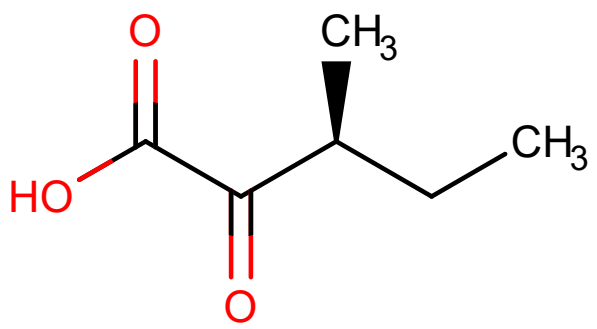	HMDB0000491	$C_6H_{10}O_3$	0.89 t (J = 7.1 Hz)	CH_3
					1.10 d (J = 6.9 Hz)	CH_3

Peak assignment	Metabolite name (*MSI metabolite identity)	Chemical structure	HMDB ID	Chemical formula	δ (ppm), multiplicity (J coupling)	Chemical group
8	2-Oxoisocaproic acid (level 1)	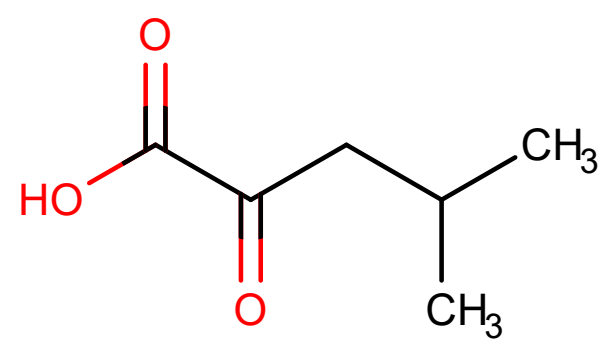	HMDB0000695	$C_6H_{10}O_3$	0.94 d (J = 6.3 Hz)	CH_3
					2.62 d (J = 6.8 Hz)	CH_3
9	Propionic acid (level 1)	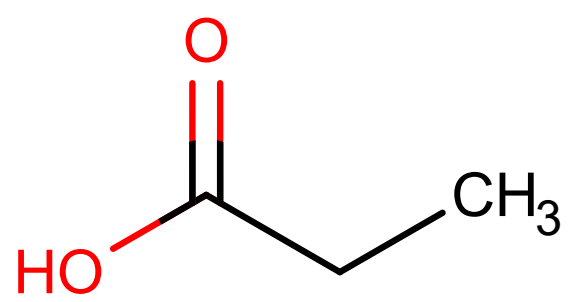	HMDB0000237	$C_3H_6O_2$	1.06 t (J = 7.1 Hz)	CH_3
10	3-Hydroxyisobutyric acid (level 1)	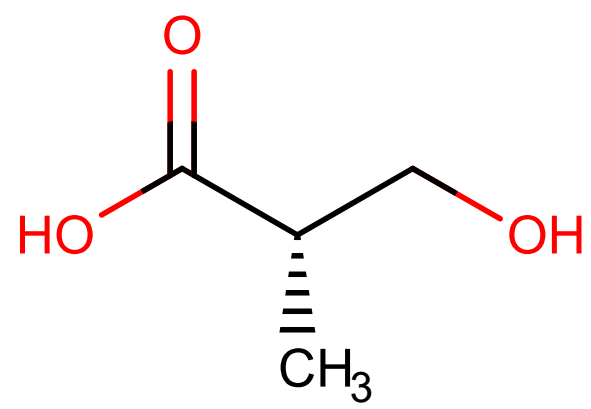	HMDB0000023	$C_4H_8O_3$	1.09 d (J = 7.0 Hz)	CH_3

Peak assignment	Metabolite name (*MSI metabolite identity)	Chemical structure	HMDB ID	Chemical formula	δ (ppm), multiplicity (J coupling)	Chemical group
11	3-Hydroxybutyric acid (level 1)	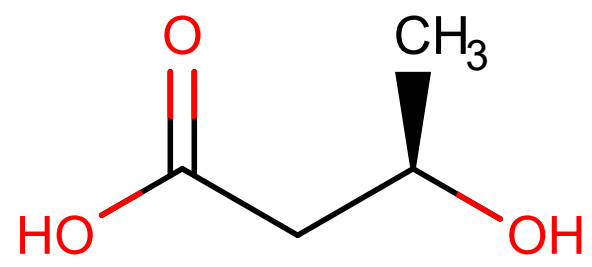	HMDB0000011	$C_4H_8O_3$	1.20 d (J = 6.2 Hz)	CH_3
					2.36 ABX (J = 8.1, 16.0 Hz)	CH_2
					4.16 m	CH
12	3-Hydroxyisovaleric acid (level 2)	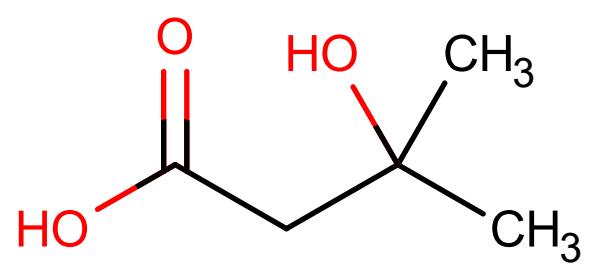	HMDB0000754	$C_5H_{10}O_3$	1.27 s	$(CH_3)_2$
					2.37 s	CH_2
13	2-Hydroxyisobutyric acid (level 2)	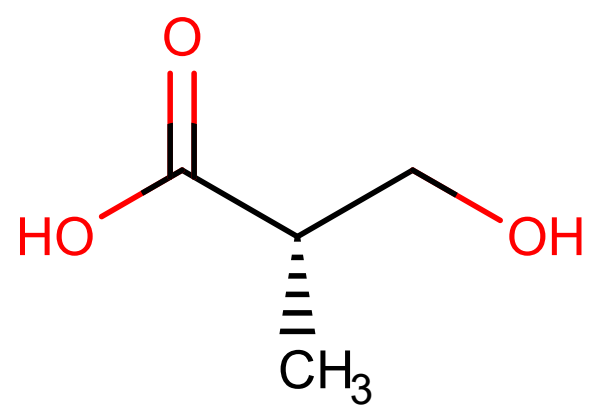	HMDB0000023	$C_4H_8O_3$	1.36 s	CH_3

Peak assignment	Metabolite name (*MSI metabolite identity)	Chemical structure	HMDB ID	Chemical formula	δ (ppm), multiplicity (J coupling)	Chemical group
14	Alanine<br>(level 1)	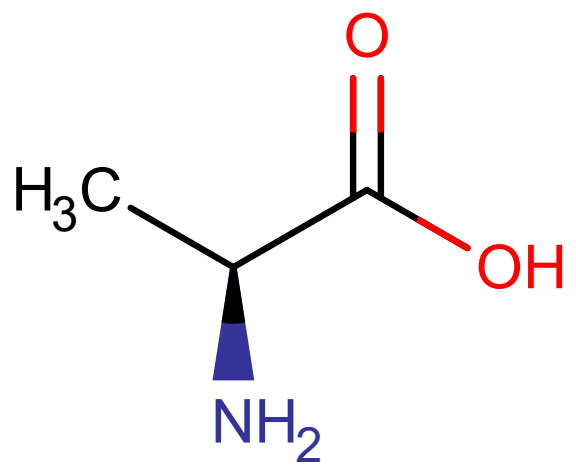	HMDB0000161	$C_3H_7NO_2$	1.48 d<br>(J = 7.3 Hz)	CH_3
15	Acetic acid<br>(level 2)	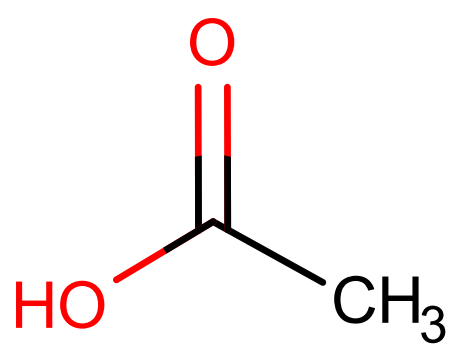	HMDB0000042	$C_2H_4O_2$	1.92 s	CH_3
16	N-Acetylglucosamine<br>(level 1)	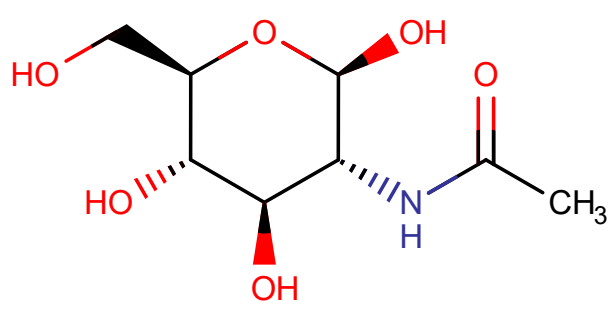	HMDB0000803	$C_8H_{15}NO_6$	1.99 s	CH_3

Peak assignment	Metabolite name (*MSI metabolite identity)	Chemical structure	HMDB ID	Chemical formula	δ (ppm), multiplicity (J coupling)	Chemical group
17	Proline (level 1)	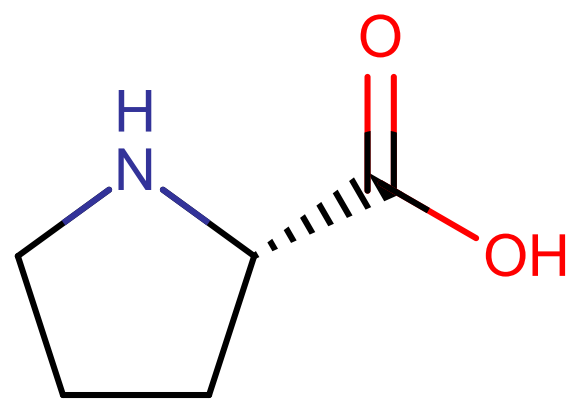	HMDB0000162	$C_5H_9NO_2$	2.00 m	CH_2
					2.30 m	CH_2
18	Methionine (level 1)	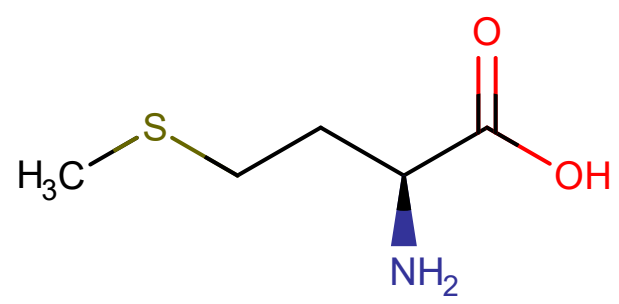	HMDB0000696	$C_5H_{11}NO_2S$	2.17 s	CH_3
					2.43 t (J = 6.4 Hz)	CH_2
19	Glutamic acid (level 1)	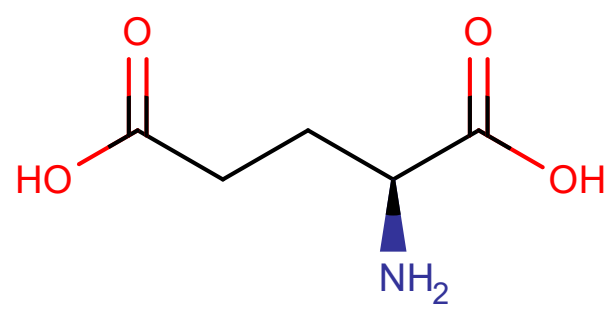	HMDB0000148	$C_5H_9NO_4$	2.05 m	CH_2
					2.35 m	CH_2

Peak assignment	Metabolite name (*MSI metabolite identity)	Chemical structure	HMDB ID	Chemical formula	δ (ppm), multiplicity (J coupling)	Chemical group
20	Glutamine (level 1)	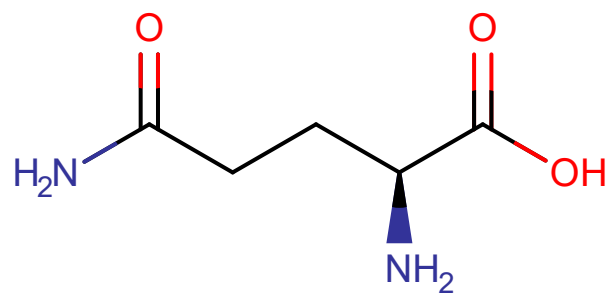	HMDB0000641	$C_5H_{10}N_2O_3$	2.16 m	CH_2
					2.43 m	CH_2
21	Acetone (level 2)	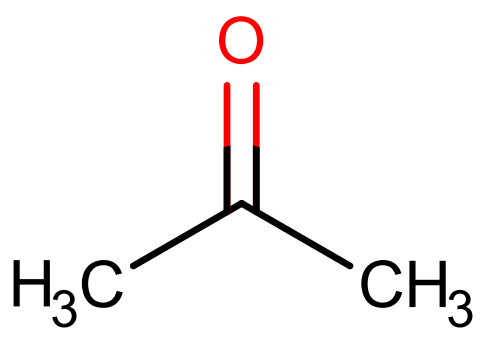	HMDB0001659	C_3H_6O	2.23 s	$(CH_3)_2$
22	Acetoacetic acid (level 2)	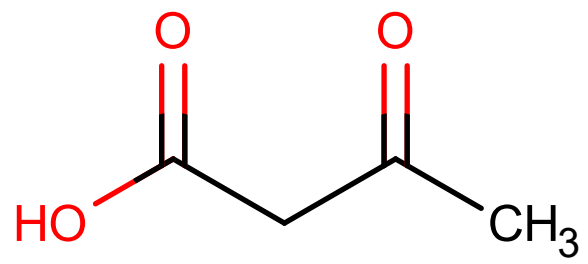	HMDB0000060	$C_4H_6O_3$	2.28 s	CH_3

Peak assignment	Metabolite name (*MSI metabolite identity)	Chemical structure	HMDB ID	Chemical formula	δ (ppm), multiplicity (J coupling)	Chemical group
23	Pyruvic acid (level 2)	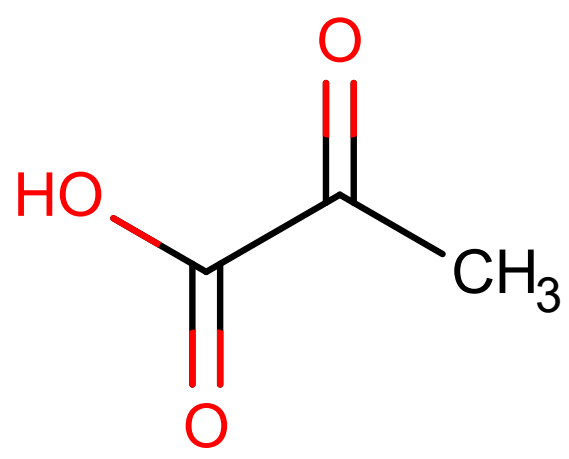	HMDB0000243	$C_3H_4O_3$	2.38 s	CH_3
24	Succinic acid (level 2)	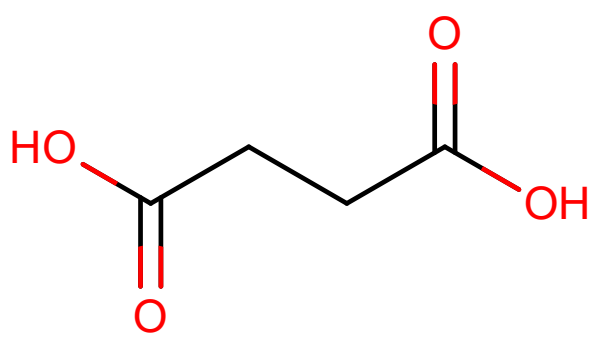	HMDB0000254	$C_4H_6O_4$	2.40 s	$(CH_2)_2$
25	Citric acid (level 1)	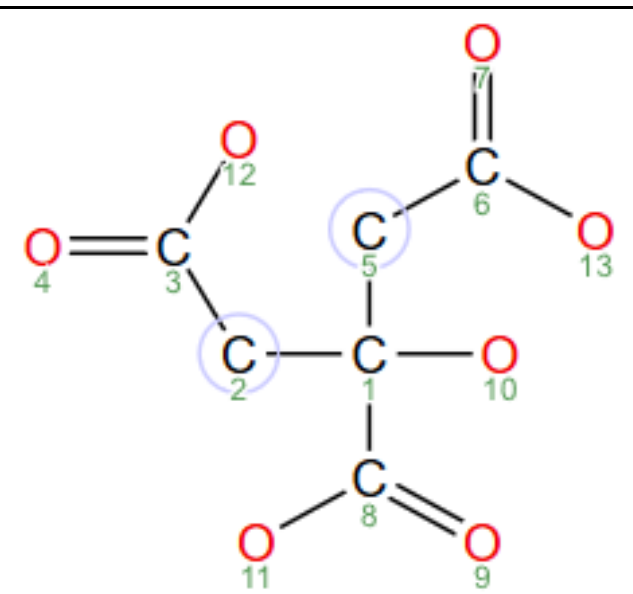	HMDB0000094	$C_6H_8O_7$	2.59 AB (J = 15.3 Hz)	$(CH_2)_2$

Peak assignment	Metabolite name (*MSI metabolite identity)	Chemical structure	HMDB ID	Chemical formula	δ (ppm), multiplicity (J coupling)	Chemical group
26	Methylamine (level 2)	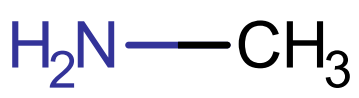	HMDB0000164	CH_5N	2.61 s	CH_3
27	Aspartic acid (level 1)	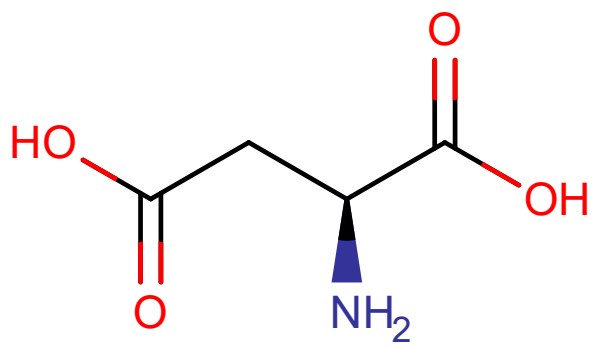	HMDB0000191	$C_4H_7NO_4$	2.68 dd (J = ND)	CH_2
					2.81 dd (J = ND)	CH_2
28	Ca-EDTA (level 1)	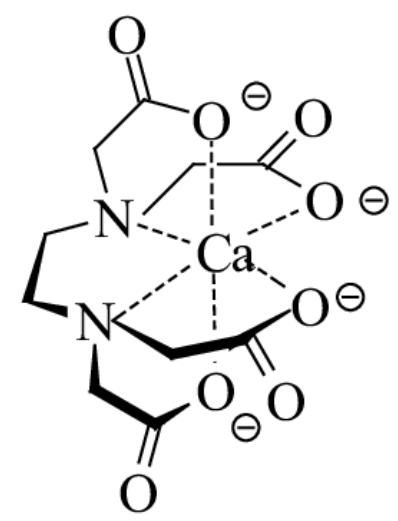	N/A	$C_{10}H_{12}CaN_2O_8$	2.56 s	$(CH_2)_2$
					3.12 q (J = 30.2, 16 Hz)	$(CH_2)_4$

Peak assignment	Metabolite name (*MSI metabolite identity)	Chemical structure	HMDB ID	Chemical formula	δ (ppm), multiplicity (J coupling)	Chemical group
29	Mg-EDTA (level 1)	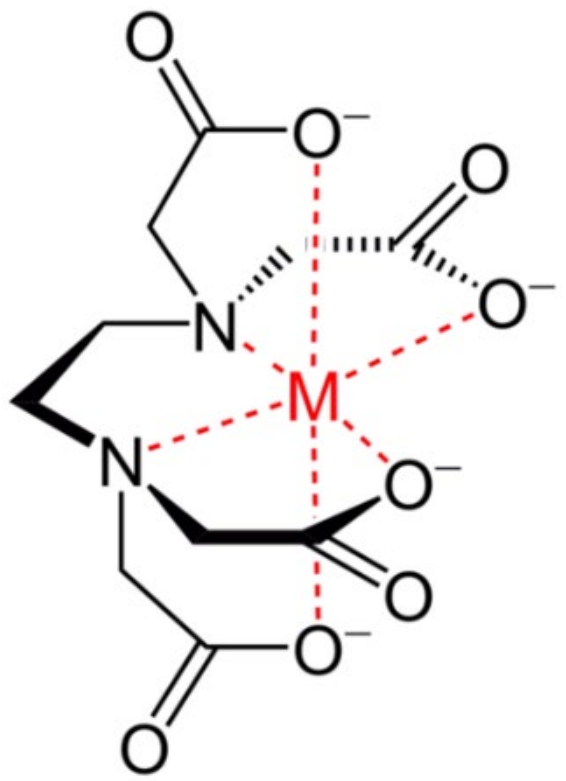	N/A	$C_{10}H_{12}MgN_2O_8$	2.70 s	$(CH_2)_2$
30	Dimethylamine (level 2)	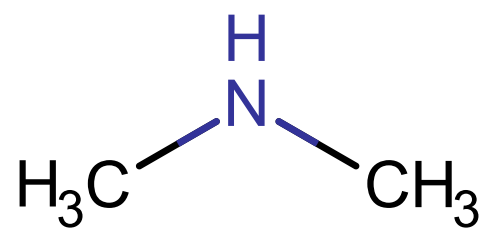	HMDB0000087	C_2H_7N	2.72 s	$(CH_3)_2$
31	Sarcosine (level 2)	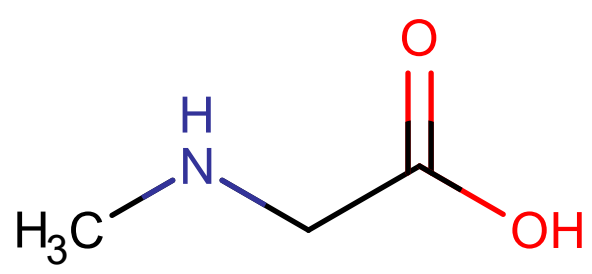	HMDB0000271	$C_3H_7NO_2$	2.72 s	CH_3

Peak assignment	Metabolite name (*MSI metabolite identity)	Chemical structure	HMDB ID	Chemical formula	δ (ppm), multiplicity (J coupling)	Chemical group
32	N,N-Dimethylformamide (level 2)	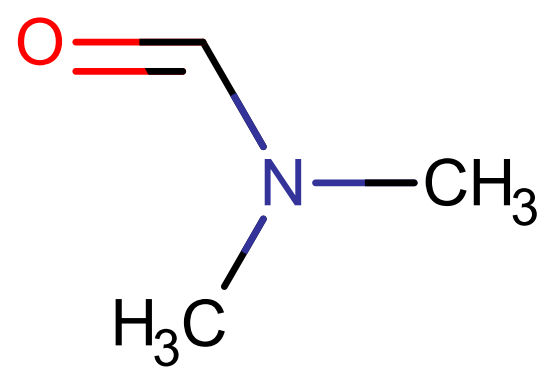	HMDB0001888	C_3H_7NO	2.86 s<br>3.02 s	CH_3<br>CH_3
33	Trimethylamine (level 2)	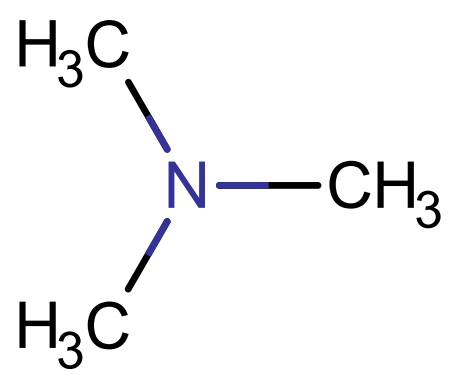	HMDB0000906	C_3H_9N	2.87 s	$(CH_3)_3$
34	N,N-Dimethylglycine (level 2)	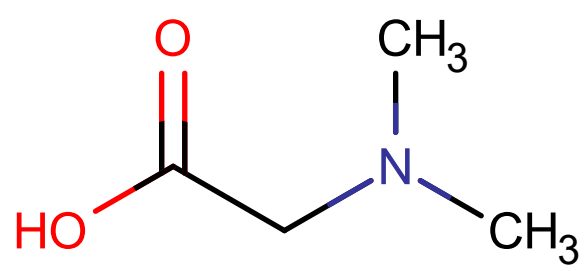	HMDB0000092	$C_4H_9NO_2$	2.93 s	$(CH_3)_2$

Peak assignment	Metabolite name (*MSI metabolite identity)	Chemical structure	HMDB ID	Chemical formula	δ (ppm), multiplicity (J coupling)	Chemical group
35	Lysine (level 1)	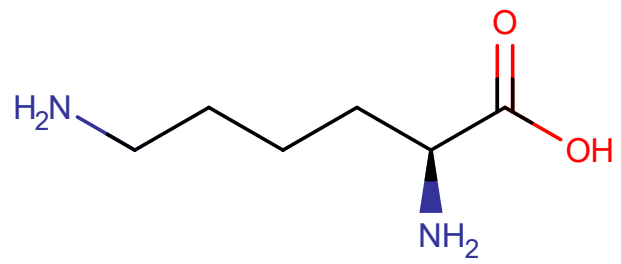	HMDB0000182	$C_6H_{14}N_2O_2$	1.73 m	CH_2
					1.91 m	CH_2
					3.03 t (J = 7.8 Hz)	CH_2
36	Creatine (level 2)	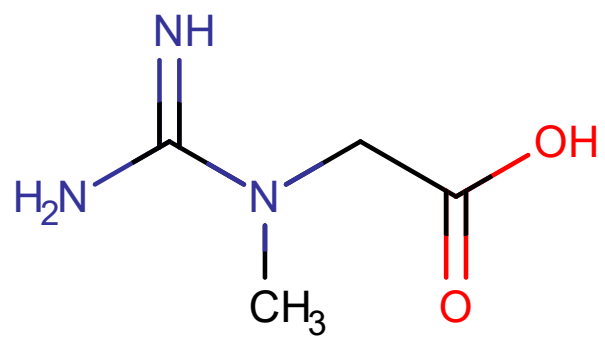	HMDB0000064	$C_4H_9N_3O_2$	3.04 s	CH_3
					3.93 s	CH_2
37	Creatinine (level 2)	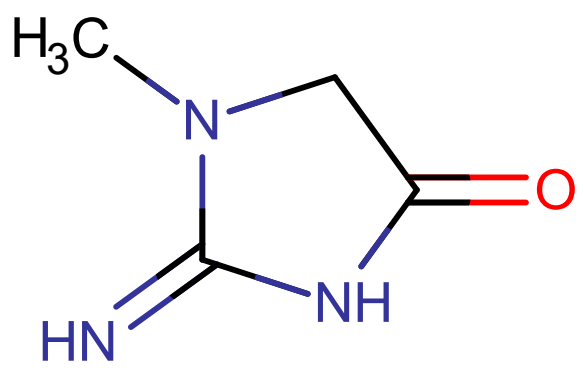	HMDB0000562	$C_4H_7N_3O$	3.045 s	CH_3
					4.06 s	CH_2

Peak assignment	Metabolite name (*MSI metabolite identity)	Chemical structure	HMDB ID	Chemical formula	δ (ppm), multiplicity (J coupling)	Chemical group
38	Ornithine (level 1)	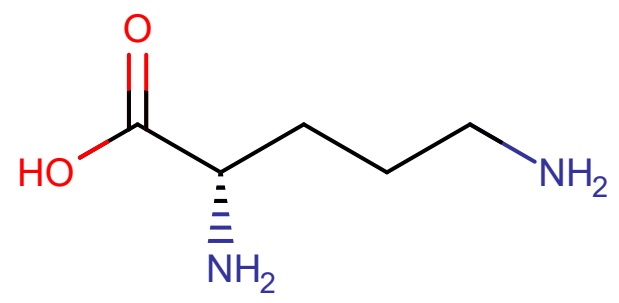	HMDB0000214	$C_5H_{12}N_2O_2$	3.06 t (J = ND)	CH_2
39	Dimethyl sulfone (level 2)	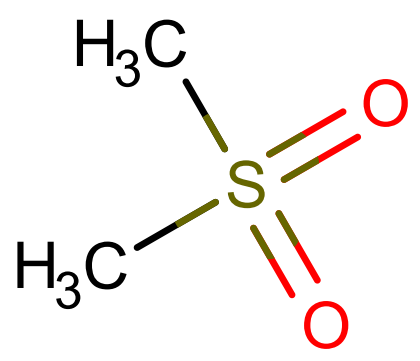	HMDB0004983	$C_2H_6O_2S$	3.16 s	$(CH_3)_2$
40	Choline (level 1)	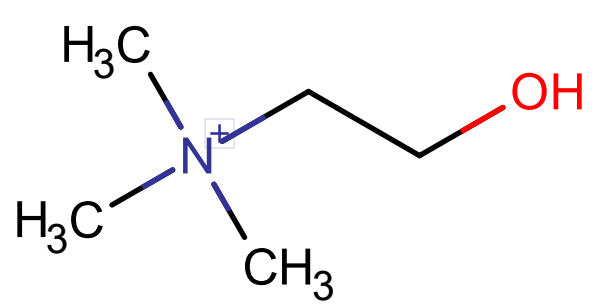	HMDB0000097	$C_5H_{14}NO$	3.20 s	$(CH_3)_3$
					4.03 t (J = 5.4 Hz)	CH_2

Peak assignment	Metabolite name (*MSI metabolite identity)	Chemical structure	HMDB ID	Chemical formula	δ (ppm), multiplicity (J coupling)	Chemical group
41	Methanol (level 2)	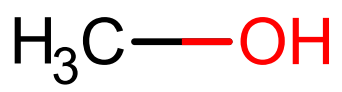	HMDB0001875	CH_4O	3.36 s	CH_3
42	Glycine (level 2)	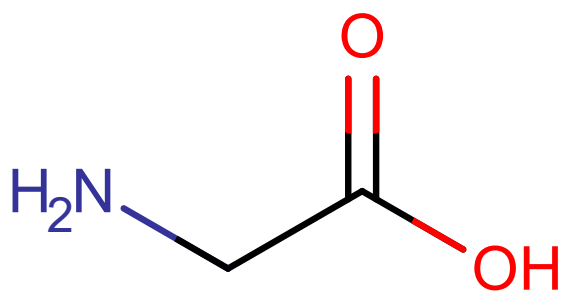	HMDB0000123	$C_2H_5NO_2$	3.57 s	CH_2
43	Glycolic acid (level 2)	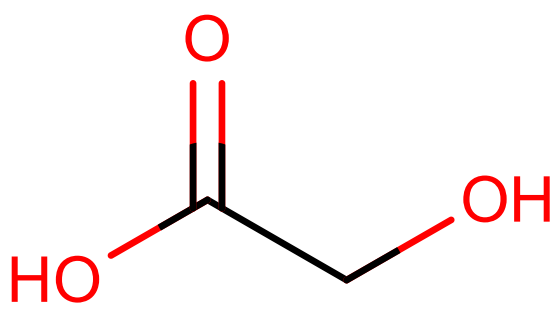	HMDB0000115	$C_2H_4O_3$	3.96 s	CH_2

Peak assignment	Metabolite name (*MSI metabolite identity)	Chemical structure	HMDB ID	Chemical formula	δ (ppm), multiplicity (J coupling)	Chemical group
44	myo-Inositol (level 1)	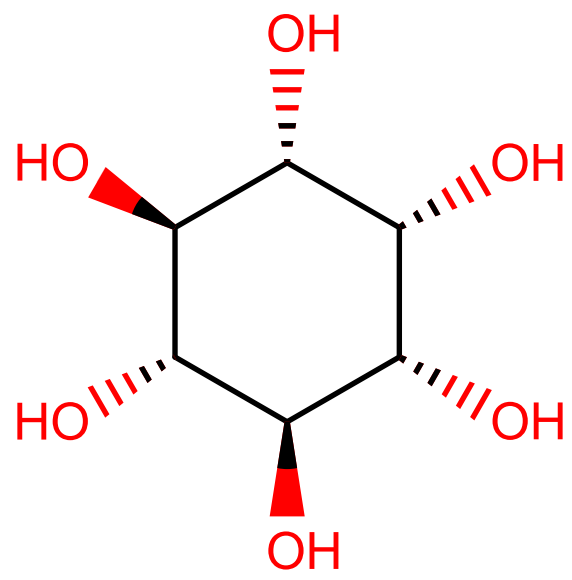	HMDB0000211	$C_6H_{12}O_6$	3.34 dd (J = ND)	CH
					4.07 t (J = ND)	CH
45	Threonine (level 1)	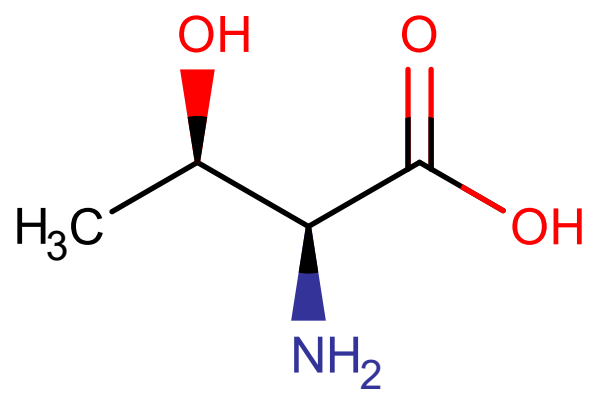	HMDB0000167	$C_4H_9NO_3$	1.32 d (J = ND)	CH_3
					4.25 q (J = ND)	CH
46	Mannose (level 1)	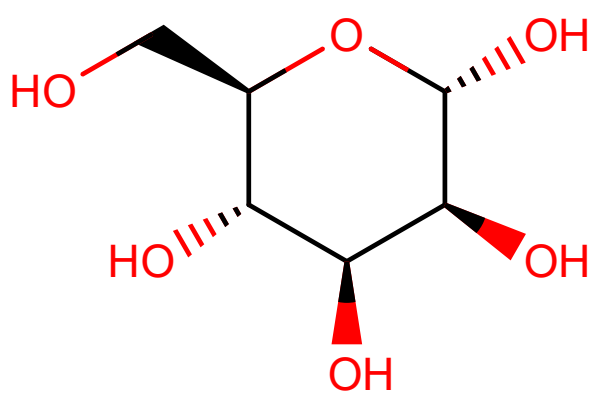	HMDB0000169	$C_6H_{12}O_6$	5.19 d (J = ND)	CH

Peak assignment	Metabolite name (*MSI metabolite identity)	Chemical structure	HMDB ID	Chemical formula	δ (ppm), multiplicity (J coupling)	Chemical group
47	Allantoin (level 2)	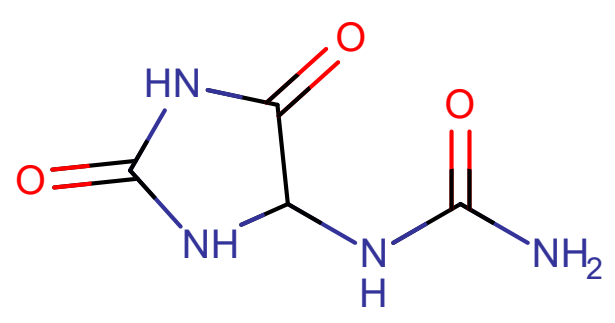	HMDB0000462	$C_4H_6N_4O_3$	5.40 s	CH
48	Fumaric acid (level 2)	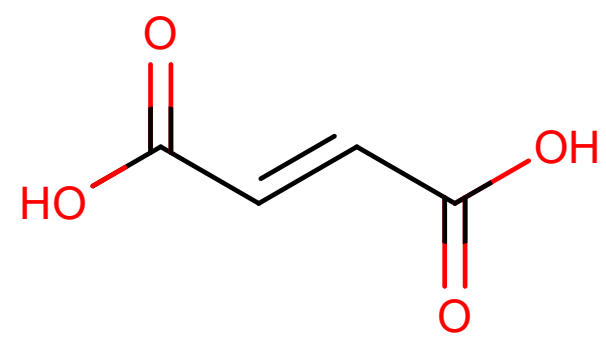	HMDB0000134	$C_4H_4O_4$	6.52 s	$(CH)_2$
49	Tyrosine (level 1)	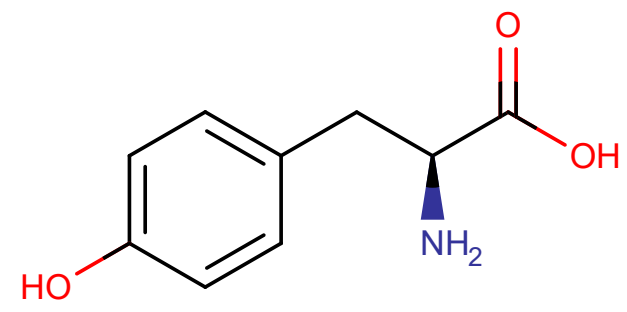	HMDB0000158	$C_9H_{11}NO_3$	6.90 dd (J = 6.6 Hz)	$(CH)_2$
					7.19 dd (J = 6.6 Hz)	$(CH)_2$

**Peak assignment**	**Metabolite name (*MSI metabolite identity)**	**Chemical structure**	**HMDB ID**	**Chemical formula**	**δ (ppm), multiplicity (J coupling)**	**Chemical group**
50	Histamine (level 2)	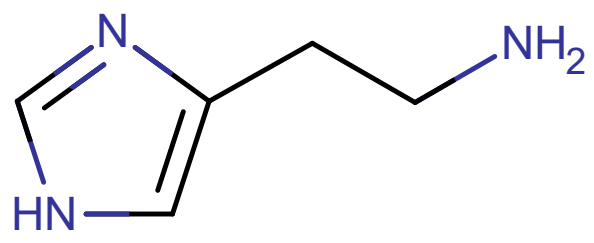	HMDB0000870	$C_5H_9N_3$	7.00 d (J = ND)	CH
51	Histidine (level 2)	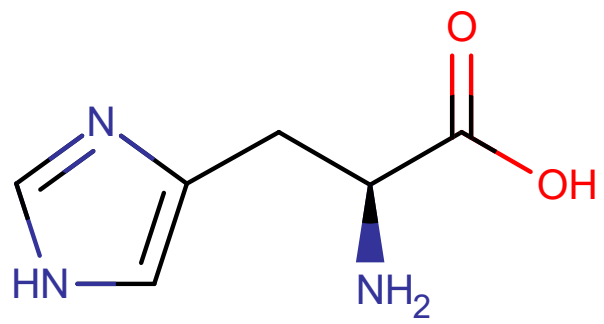	HMDB0000177	$C_6H_9N_3O_2$	7.07 d (J = ND)	CH
					7.80 d (J = ND)	CH
52	Phenylalanine (level 1)	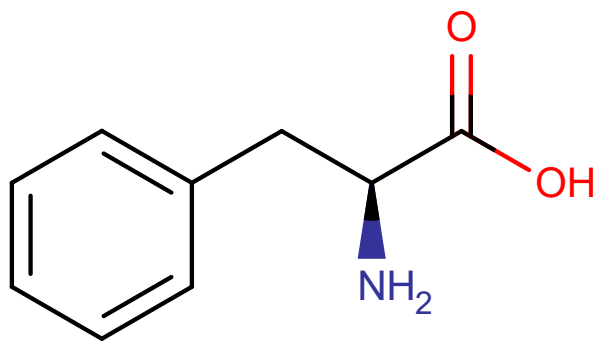	HMDB0000159	$C_9H_{11}NO_2$	7.39 m	$(CH)_5$

Peak assignment	Metabolite name (*MSI metabolite identity)	Chemical structure	HMDB ID	Chemical formula	δ (ppm), multiplicity (J coupling)	Chemical group
53	Hippuric acid (level 1)	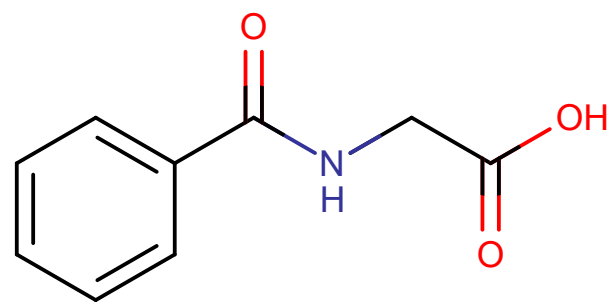	HMDB0000714	$C_9H_9NO_3$	7.55 dd (J = 7.7 Hz)	$(CH)_2$
					7.64 tt (J = 7.7 Hz)	$(CH)_2$
					7.83 dd (J = 7.4, 1.5 Hz)	CH
54	Formic acid (level 2)	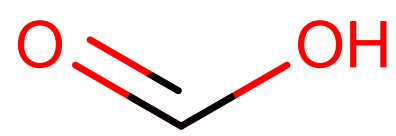	HMDB0000142	CH_2O_2	8.46 s	CH
Glc	Glucose (level 1)	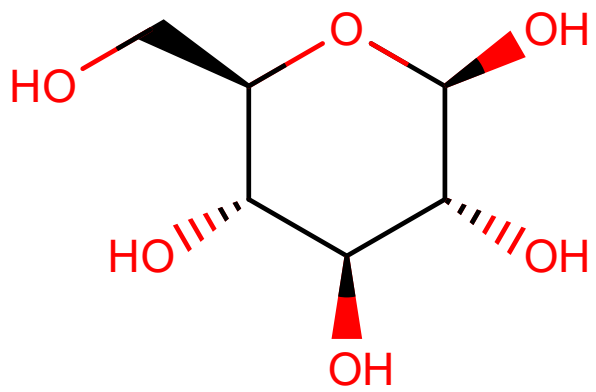	HMDB0000122	$C_6H_{12}O_6$	3.22-3.27	
					3.38-3.92	
					4.65 d (J = 8.3 Hz)	CH
					5.24 d (J = 3.5 Hz)	CH

Peak assignment	Metabolite name (*MSI metabolite identity)	Chemical structure	HMDB ID	Chemical formula	δ (ppm), multiplicity (J coupling)	Chemical group
LA	Lactic acid (level 1)	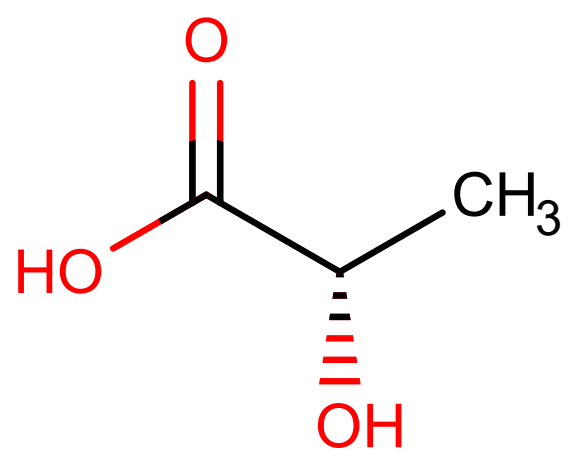	HMDB0000190	$C_3H_6O_3$	1.33 d (J = 7.0 Hz)	CH_3
					4.12 q (J = 7.0 Hz)	CH
EDTA	Ethylenediaminetetraacetic acid (level 2)	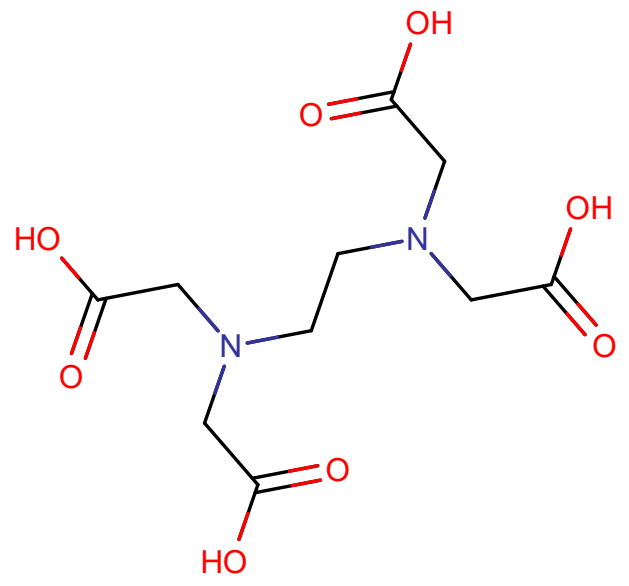	HMDB0015109	$C_{10}H_{16}N_2O_8$	3.22 s	$(CH_2)_2$
					3.62 s	$(CH_2)_4$
IS	TSP	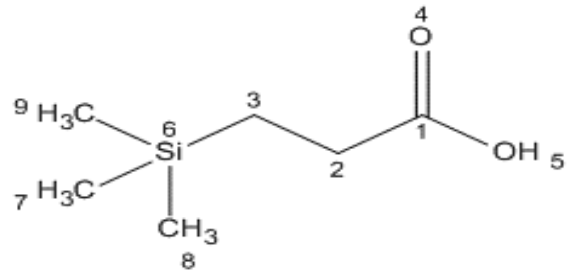	N/A	$C_6H_{14}O_2Si$	0.00 s	Si-$(CH_3)_3$

***Metabolomics Standards Initiative (MSI) metabolite identity: level 1 = confirmed identity by reference standards (confirmed using 2D COSY NMR); level 2 = putatively annotated (confirmed 1D but cannot confirm using 2D COSY NMR [e.g., singlet(s)]).**
