# Supplementary material for: Comparative 1H-NMR profiling of plasma and serum metabolites in giraffes and white rhinoceroses
Source: Metabolomics. 2026 Sep 23;22(5):159. doi: 10.1007/s11306-026-02535-0 (PMC13601079; doi:10.1007/s11306-026-02535-0)
Supplement: Supplementary file 2 [file 11306_2026_2535_MOESM2_ESM.pdf]

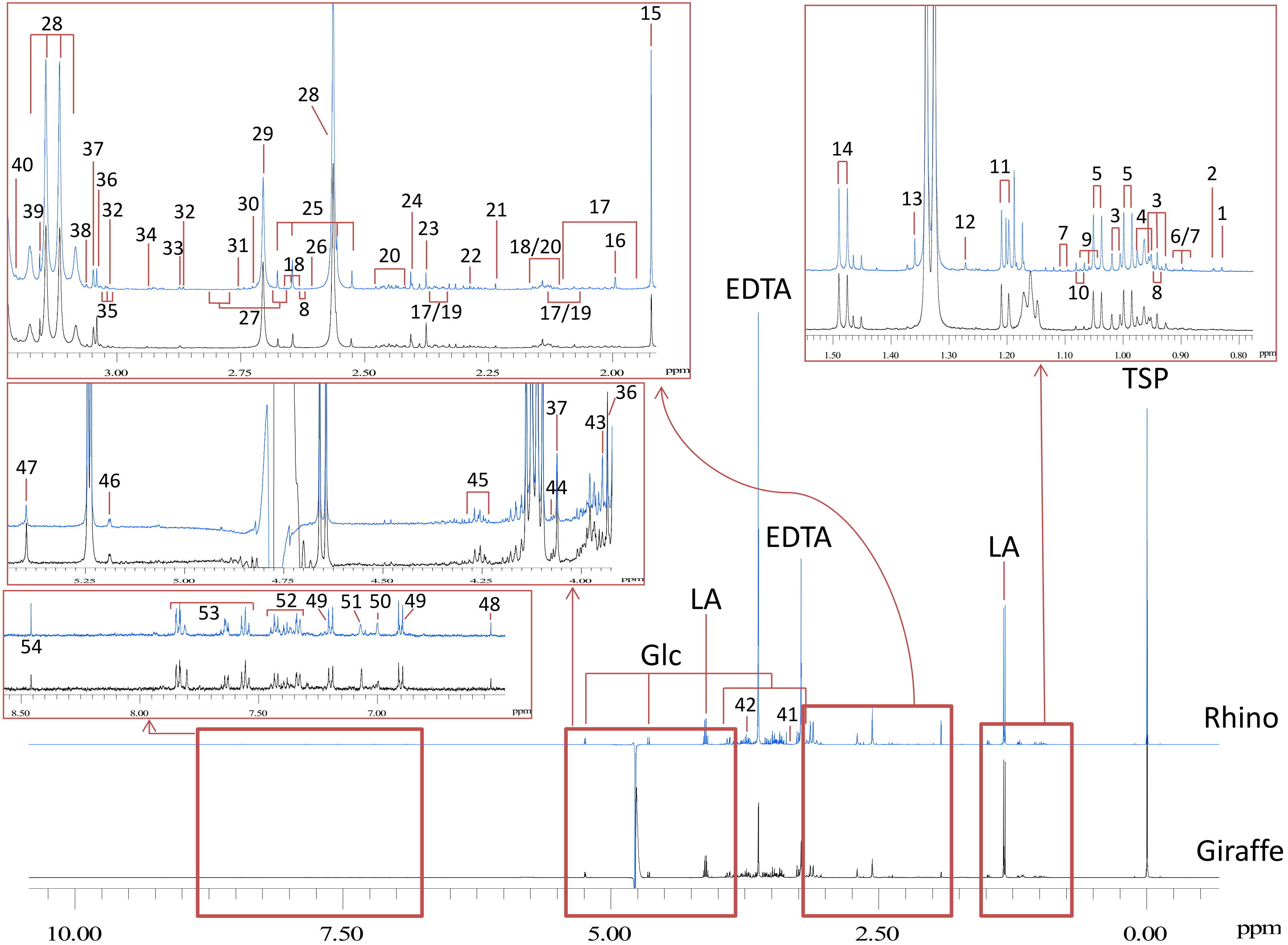

Rhino
Giraffe
EDTA
EDTA
TSP
LA
LA
Glc
42
41
10.00
7.50
5.00
2.50
0.00
ppm

- 1 = cholic acid; 2 = glycocholic acid; 3 = isoleucine; 4 = leucine; 5 = valine; 6 = 2-hydroxybutyric acid; 7 = 3-methyl-2-oxovaleric acid; 8 = 2-oxoisocaproic acid; 9 = propionic acid; 10 = 3-hydroxyisobutyric acid; 11 = 3-hydroxybutyric acid; 12 = 3-hydroxyisovaleric acid; 13 = 2-hydroxyisobutyric acid; 14 = alanine; 15 = acetic acid; 16 = N-acetylglucosamine; 17 = proline; 18 = methionine; 19 = glutamic acid; 20 = glutamine; 21 = acetone; 22 = acetoacetic acid; 23 = pyruvic acid; 24 = succinic acid; 25 = citric acid; 26 = methylamine; 27 = aspartic acid; 28 = Ca-EDTA; 29 = Mg-EDTA; 30 = dimethylamine; 31 = sarcosine; 32 = N,N-dimethylformamide; 33 = trimethylamine; 34 = N,N-dimethylglycine; 35 = lysine; 36 = creatine; 37 = creatinine; 38 = ornithine; 39 = dimethyl sulfone; 40 = choline; 41 = methanol; 42 = glycine; 43 = glycolic acid; 44 = myo-inositol; 45 = threonine; 46 = mannose; 47 = allantoin; 48 = fumaric acid; 49 = tyrosine; 50 = histamine; 51 = histidine; 52 = phenylalanine; 53 = hippuric acid; 54 = formic acid.
- Glc = glucose; LA = lactic acid; EDTA = ethylenediaminetetraacetic acid; TSP = trimethylsilylpropanoic acid (IS).
